# Improving diabetic retinopathy screening at the point of care: integrating telemedicine to overcome current challenges

**DOI:** 10.1186/s12886-024-03508-4

**Published:** 2024-06-14

**Authors:** Farinaz Salavatian, Nahid Hashemi-Madani, Zahra Emami, Zahra Hosseini, Khalil Ghasemi Falavarjani, Mohammad E. Khamseh

**Affiliations:** 1 Tehran, Iran; 2 Endocrine Research Center, Institute of Endocrinology and Metabolism, Iran University of Medical Sciences, Tehran, Iran, No. 10, Firoozeh St., Vali-asr Ave., Vali-asr Sq, Tehran, Iran; 3 Cardiovascular Intervention Research Center, Rajaie Cardiovascular Medical and Research Center, Iran University of Medical Sciences, Tehran, Iran; 4 Eye Research Centre, Five Senses Health Institute, School of Medicine, Hazrat Rasoul Akram Hospital, Iran University of Medical Sciences, Tehran, Iran, Sattarkhan St., Niayesh St, Tehran, 14455-364 Iran

**Keywords:** Diabetes retinopathy, Fundus photography, Screening rate, Adherence rate

## Abstract

**Objective:**

To investigate the utility of point of care screening of diabetic retinopathy (DR) and the impact of a telemedicine program to overcome current challenges.

**Methods:**

This was a retrospective study on people with type 2 diabetes mellitus (T2DM) who were screened for DR using the single-field non-mydriatic fundus photography at the point of care during routine follow-up visits at endocrinology clinic. Retinal images were uploaded and sent to a retina specialist for review. Reports indicating retinopathy status and the need for direct retinal examination were transmitted back to the endocrinology clinic. All patients were informed about DR status and, if needed, referred to the retina specialist for direct retinal examination.

**Results:**

Of the 1159 individuals screened for DR, 417 persons (35.98%) were screen-positive and referred to the retina specialist for direct retinal examination. A total of 121 individuals (29.01%) underwent direct retinal examination by the specialist. Diabetes macular edema (DME) was detected in 12.1%. In addition, non-proliferative diabetic retinopathy (NPDR) and proliferative diabetic retinopathy (PDR) were detected in 53.4% and 2.6% of the patients, respectively.

**Conclusion:**

Integrating DR screening program at the point of care at the secondary care services improves the rate of DR screening as well as detection of sight threatening retinopathy and provides the opportunity for timely intervention in order to prevent advanced retinopathy in people with T2DM.

## Introduction

Diabetic retinopathy (DR) is a common micro-vascular complication of diabetes affecting more than 100 million individuals globally and is a leading cause of blindness especially among the working-age adult people [1, 2]. Additionally, it is expected that the global prevalence of DR increases significantly from about 103 million people in 2020, to 130 million in 2030, and 160 million in 2045 [3]. The global increasing in the prevalence of diabetes, lifestyle changes, and increasing lifespan might explain this projection [3]. The rates of increase in DR prevalence is higher in low-and-middle- income regions with the largest increase is expected to occur in the Middle East and North Africa (MENA) [3]. Thus, a comprehensive DR screening program to target people with T2DM is an urgent need.

Various methods are being used to screen DR including ocular examination by an ophthalmologist, and imaging techniques such as color fundus photography and optical coherence tomography (OCT) [4]. They have their own advantages and disadvantages. Although the standard method for screening DR is clinical ocular examination, the health economic burden associated with referral of the patients is a major concern. Retinal photography provides adequate field of retina for examination and can be used by trained technicians. The images can be stored for a long time; and can be examined at the different times by various professionals [4]. Although the single-field fundus photography is not a substitute for a comprehensive examination, it is of acceptable accuracy as a screening tool to identify patients with retinopathy [5, 6]. American diabetes association (ADA) recommends programs using retinal photography to improve access to DR screening [7]. On the other hand, to increase the effectiveness of the screening program we also require a standard screening algorithm. In this study, we described a DR screening program at a secondary care service using single-field fundus photography at the point of care using telemedicine and in collaboration with retina specialist.

## Methods

This study retrospectively included known cases of type two diabetic people referred to an endocrinology clinic between 2016 and 2019 for their routine follow-up visits; and fulfilled the ADA criteria for DR screening [1]. The study protocol was approved by the Ethics Committee (EC) of the Institutional Review Board of the Iran University of Medical Science; ethics code: IR.IUMS.REC.1402.1081. The eligible participants were screened using the single-field non-mydriatic fundus photography obtained with a retinal camera (AFC-330 Non-mydriatic Auto Fundus Camera, NIDEK Inc. Japan). The camera takes 45 degrees color fundus retinal images centered on the fovea. The anonymous retinal images were sent to a retina specialist using a social media app (Telegram Messenger). All the images were interpreted and graded based on International Classification of Diabetic Retinopathy scale [8]. In brief, images with no or mild non-proliferative diabetic retinopathy (NPDR) were considered as screen-negative (non-referable) images, whereas those with moderate to severe NPDR or worse were classified as screen-positive (referable). Vision-threatening diabetic eye disease (VtDED) was defined as proliferative diabetic retinopathy (PDR) or the presence of diabetic macular edema (DME) [8]. Reports indicating retinopathy status and the need for direct retina examination were transmitted back to the endocrinology clinic. All patients were informed of their screening results and received the standard education about the consequences of DR. The screen-positive (referable) patients were referred to the retina specialist for direct retinal examination. Rate of attendance at the specialist visit was calculated by reviewing the ophthalmology clinic records. Moreover, the results of direct retinal examination were assessed.

### Statistical analysis

The data were analyzed using STATA software. The continuous variables were described using mean (standard deviation (SD)) and median (interquartile range (IQR)), and t-test and Mann-Whitney test were the tools of inferring the data, respectively. The discrete variables were reported using number (percent) and the Chi-squared test was used for them. Considering the binary nature of the response variable, the logistic regression models were fitted on the covariates, in crude and adjusted versions. These models led to reporting odds ratios (ORs). P value less than 0.05 was considered significant.

## Results

**Fig. 1:**
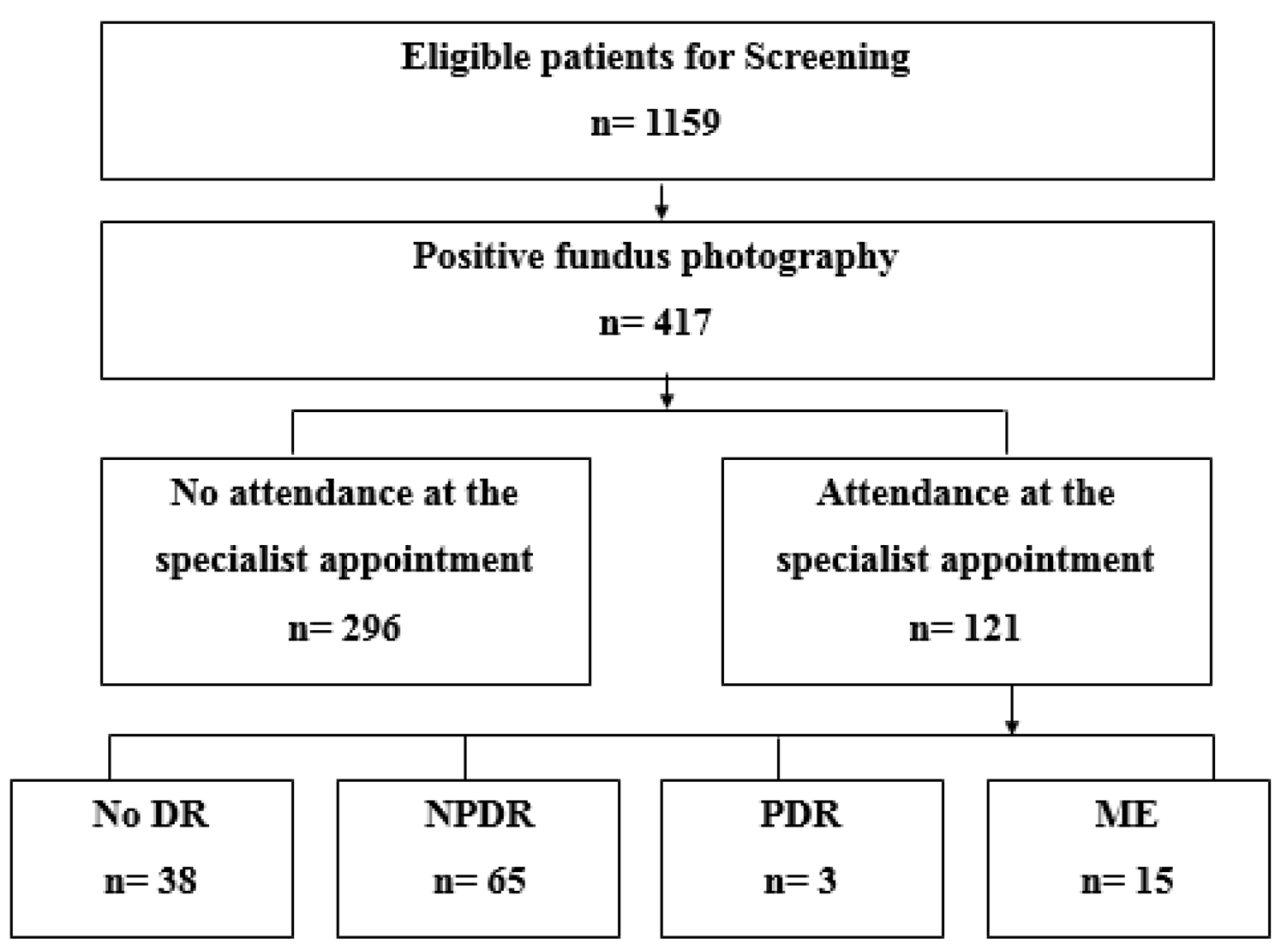
Flow diagram to demonstrate the participants at the point of care DR screening program NPDR; non-proliferative diabetic retinopathy, PDR; proliferative diabetic retinopathy, ME; macular edema

This study included 1159 patients with T2DM who were screened for DR using the single-field non-mydriatic fundus photography. Based on the interpretation of the images, 417 individuals (35.98%) considered as screen-positive patients and were asked to refer to the retina specialist for direct retinal examination. One hundred twenty-one individuals (29.01%) were visited by the retina specialist. The mean interval between the referral date and attendance at the retina specialist visit was 4.9 (± 8.9) months. Direct retinal examination of the 121 individuals indicated no DR in 31.9% and NPDR in 53.4% of the patients. Moreover, PDR and ME were reported in 2.6% and 12.1%, respectively (Fig. 1). There were no missing data regarding the included patients.

Characteristics of the patients screened for DR are presented in Table 1. Compared to the patients considered as screen-negative (non-referable), those considered as screen-positive patients (referable) were significantly older (56.6 vs. 53.9 years; *p* < 0.001), had longer duration of diabetes (8 vs. 5 years; *p* < 0.001), and had higher systolic blood pressure (SBP) (126.3 vs. 123.7 mmHg; *p* = 0.048. There was no significant difference considering glycated hemoglobin (HbA1C), diastolic blood pressure (DBP), or percentage of insulin users between the two groups.

**Table 1:** Baseline characteristics of the participants

	Screen-Negative patients (*N*: 742)	Screen-Positive patients (*N*: 417)	*P*-value
Age (yrs.)*	53.9 (12.1)	56.6 (12.7)	< 0.001
Sex (% male) †	375 (51.4%)	198 (47.6%)	0.219
Duration of diabetes (yrs)**	5 (3–10)	8 (4–14)	< 0.001
HbA1C (%)*	7.95 (1.85)	8.03 (1.87)	0.541
SBP (mmHg)*	123.7 (18.5)	126.3 (20.4)	0.048
DBP (mmHg)*	77.2 (30.3)	75.8 (10.6)	0.425
Insulin users (%)†	301 (41.0%)	172 (41.3%)	0.937

Data are presented as mean (SD)*, median (IQR)**, or number (percentage)†. HbA1C; glycated hemoglobin, SBP; systolic blood pressure, DBP; diastolic blood pressure

Characteristics of screen-positive patients are presented in Table 2. Compared to the patients who did not attend at the retina specialist appointment, those who attended at the retina specialist appointment had a longer duration of diabetes (10 vs. 7 years; *p* = 0.001), and higher SBP (130.2 vs. 124.8 mmHg; *p* = 0.03). They also were more likely to use insulin (49.6 vs. 37.8%; *p* = 0.02). Moreover, 15 individuals (12.4%) of the patients who attended at the retina specialist appointment were referred due to VtDED.

**Table 2:** Characteristics of the screen-positive patients based on attendance at retina specialist visit

	No attendance at the retina specialist appointment (*N*: 296)	Attendance at the retina specialist appointment (*N*: 121)	*p*- value
Age (yrs)*	55.8 (12.9)	58.5 (11.8)	0.051
Sex (% male) †	147 (49.8%)	51 (42.2%)	0.154
Duration of diabetes (yrs)**	7 (3–12)	10 (5–15)	0.001
HbA1C (%)*	8.06 (1.90)	7.96 (1.80)	0.682
SBP (mmHg) *	124.8 (19.4)	130.2 (22.6)	0.030
DBP (mmHg)*	76.1 (10.6)	75.1 (10.4)	0.455
Insulin users (%)†	112 (37.8%)	60 (49.6%)	0.027
VtDED (%)†	0 (0%)	15 (12.4%)	< 0.001

Data are presented as mean (SD)*8, median (IQR)**, or number (percentage)†. HbA1C; glycated hemoglobin, SBP; systolic blood pressure, DBP; diastolic blood pressure, VtDED; vision threatening diabetic eye disease, DR: diabetic retinopathy

We further applied regression logistic models to determine factors associated with attendance at the retina specialist appointment. Although crude model indicated age, duration of diabetes, SBP, and insulin use were associated with greater odds of attendance at the retina specialist visit, the results were not significant in the adjusted model (Table 3).

**Table 3:** Factors associated with the attendance at retina specialist appointment

Variable	Crude		Adjusted	
	OR (95% CI)	p-value	OR (95% CI)	p-value
Age(yrs)	1.02 (1-1.04)	0.049	1.02 (1.00-1.05)	0.069
Duration of diabetes (yrs)	1.05 (1.02–1.08)	< 0.001	1.04 (1.00-1.07)	0.065
SBP (mmHg)	1.01 (1-1.03)	0.037	1.01 (1.00-1.02)	0.134
Insulin users (%)	1.62 (1.05–2.48)	0.028	1.42 (0.84–2.39)	0.192

SBP; systolic blood pressure

## Discussion

In this study the rate of screen-positive diabetic retinopathy using the single-field non-mydriatic fundus photography was 35.98%. These patients were referred to a retina specialist. Of whom, 29.01% attended the retina specialist appointment; and more than 14% of them were found to have VtDED (DME and PDR).

A single-center study on patients with T2DM also showed screen-positive diabetic retinopathy in 28% of the participants, using a non-mydriatic, single-field retinal photography [9]. Another multicenter cross-sectional screening study used non-mydriatic fundus photographs estimated national prevalence of diabetic retinopathy in 12.5% of Indian population [10]. However, these studies did not investigate patient adherence to the referral recommendations [9]. A nationwide study of DR screening in Brazil indicated the overall screening coverage increased from 12.1% in 2014 to 21.2% in 2019 (*p* < 0.001) with substantial discrepancies in different regions [11]. This study included the patients who underwent screening using either dilated fundus examination or color fundus photograph [11]. The investigators concluded that screening for DR in patients with diabetes is ineffective in Brazil [11]. Interestingly, color fundus photographs consisted only 9.0% of the screening procedures and most patients underwent dilated fundus examination [11].

The ADA recommendation is performing fundus photography and interpretation with remote reading or use of a validated assessment tool can be an appropriate screening strategy for DR [7]. It has been shown fundus photography has the potential to increase the screening coverage of high-risk patients for DR and to enhance the efficiency and reduce the costs especially in areas where qualified eye care professionals are not available [12, 13]. Retinal imaging techniques have considerably evolved over the recent decades. The use of ultra wide field (UWF) fundus imaging improves the detection of DR lesions and leads to precise grading of DR [14]. However, the major problem of UWF imaging is high cost and limited availability. Moreover, use of artificial intelligence (AI) can aid in quick screening of DR and minimize requirement of trained human resource [15]. Although applying fundus photography improves DR screening, a structured program is needed to provide pathways for timely referral and direct retinal examination to increase patients’ adherence.

Adherence to DR screening recommendations continues to be a challenge. We found that about one third of screen-positive patients attended the specialist appointment. Although patients who adhered to the recommendations had longer duration of diabetes and higher SBP, and were more likely to use insulin, the effect of these factors was no longer remained significant after applying multivariate regression analysis. Although we did not specifically investigate the attitude of the participants toward their own responsibility to be in charge of their screening program, the results indicated the patients’ adherence to the recommendations is more likely to be due to other factors like their personal attitude rather than disease specific characteristics [16, 17].

A large number of screen-positive patients did not attend at the ophthalmologist appointment. Lack of symptoms might be a reason why these patients missed the DR screening appointment [16]. Investigation of factors associated with adherence to the DR screening program showed that the adherent patients were more likely to have family or friends experienced diabetic-related vision loss [16]. Moreover, reminding for the appointment time is another important issue that might improve the adherence to the appointment [16]. However, the patients included in this study did not receive reminder for the ophthalmologist appointment. Furthermore, they were screened for DR at the time of their routine follow-up at the endocrinology appointment, and were not forced to visit our collaborating ophthalmologist; thus, they might refer to another ophthalmologist.

Although many studies evaluated the adherence to DR screening programs, only a few specifically explored the adherence of screen-positive patients to the follow-up recommendations. A retrospective study of 974 patients with diabetes in an academic primary care clinic indicated 33.9% were adherent to ophthalmic screening appointments within a two-year period [18]. In addition, adherence during a one-year interval following the reference visit decreased to 18.7% [18]. Compared to this study, higher percentage of our patients attended at the retina specialist appointment within the first year. This indicated point of care DR screening may improve patient’s adherence. However, it should be considered that the above-mentioned study included both type one and two patients with diabetes encouraged to refer to the eye clinic for an annual diabetic eye examination in accordance with the screening recommendations. Thus, they were not necessarily screen-positive patients.

Moreover, more holistic health care programs namely, screening and assessment of diabetic foot as well as structured diabetes education sessions were associated with a greater adherence to diabetic retinopathy screening recommendations [18]. A survey of diabetic patients with low socioeconomic status showed an annual screening rate of 55%, although majority of them received a physician recommendation for DR screening [19]. Most patients reported financial burden and depression as the main barriers [19]. Low adherence to DR screening programs is also attributed to the lack of resources and infrastructures, lack of trained eye care professionals, and suboptimal access to care [20].

Many studies proposed the solutions to improve the DR screening programs. Well-planned education programs for physicians could increase the number of referrals and attendance of the patients for DR screening [21]. Moreover, beneficial effects of patient education programs on different aspects of diabetes care including retinopathy surveillance are well-established [22, 23]. Development of local guidelines or regional programs specific to resource setting is also suggested [20, 24].

**Fig. 2:**
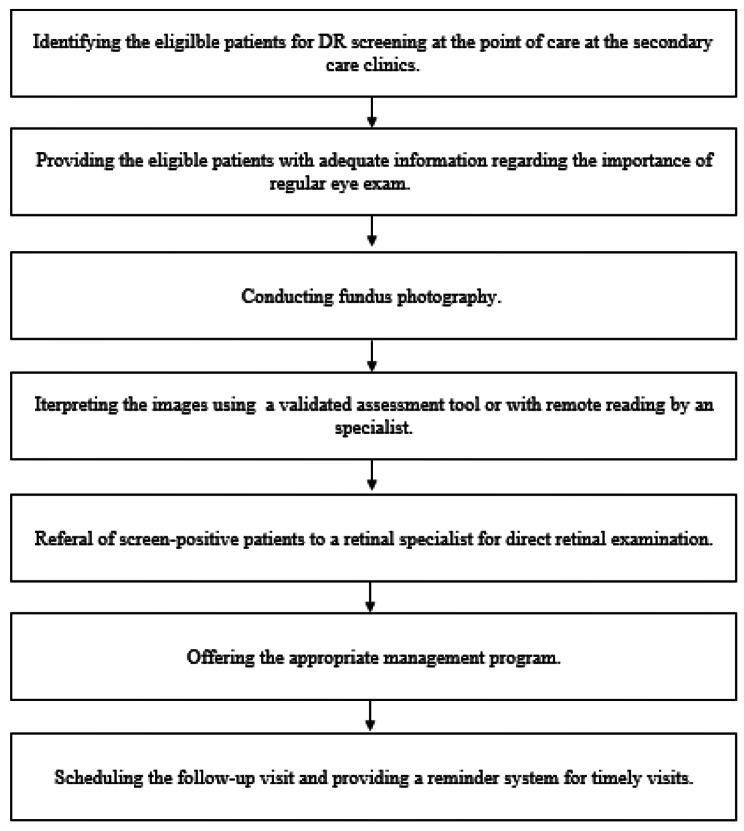
Suggested strategy for a local diabetic retinopathy screening program at the point of care

We integrated DR screening program into the routine patient care referred to the endocrinology clinic. This approach seems to increase the rate of detection of DR and DME as well as adherence to the recommendation and decrease the interval of attendance at retinal specialist visit. Similarly, a recent study showed screening of DR at the tertiary point of care services enhances detection of DR [25]. This study indicated secondary care services can also have an important role in improvement of DR screening program. Accordingly, we proposed developing a clear care pathway for DR screening of the patients referred to the endocrinologist for receiving their routine diabetes care (Fig. 2).

## Strengths and limitations

Most previous studies evaluated DR screening rate at the primary care clinics. But we assessed DR screening rate at the time of the routine care for diabetes at the secondary care services. Moreover, we focused on the adherence of the screen-positive patients, while most previous studies evaluated the adherence to the DR screening programs. The images were taken with a single retinal camera; and one retina specialist reviewed the images. However, this was a single center study and the results should be generalized with caution. Furthermore, we did not assess some important factors such as knowledge regarding DR screening, perceived risk and self-control, relationship with physician, and economic aspects, all reported to have an important role in the adherence to DR screening programs.

## Conclusion

Screening of DR at the point of care in the secondary care clinics could improve the DR screening program through including a large number of patients in the screening program. Moreover, using fundus photography technique could provide the opportunity for early detection of patients with sight threatening retinopathy. This approach could be applied in local areas in collaboration of endocrinologists with retina specialist.

## Data Availability

The datasets used and analyzed during the current study are available from the corresponding author on reasonable request.

## Abbreviations

ABI: Ankle brachial index
ADA: American diabetes association
AI: Artificial intelligence
DBP: Diastolic blood pressure
DME: Diabetic macular edema
DR: Diabetic retinopathy
EC: Ethics Committee
HbA1C: glycated hemoglobin
IQR: Interquartile range
ME: Macular edema
MENA: Middle East and North Africa
NPDR: Non-proliferative diabetic retinopathy
OCT: Optical coherence tomography
PDR: Proliferative diabetic retinopathy
SD: Standard deviation
SBP: Systolic blood pressure
T2DM: Type 2 diabetes mellitus
UWF: Ultra wide field
VtDED: Vision-threatening diabetic eye disease

## References

[1] Yau JW, Rogers SL, Kawasaki R, Lamoureux EL, Kowalski JW, Bek T (2012). Global prevalence and major risk factors of diabetic retinopathy. Diabetes Care.

[2] Ting DS, Cheung GC, Wong TY (2016). Diabetic retinopathy: global prevalence, major risk factors, screening practices and public health challenges: a review. Clin Exp Ophthalmol.

[3] Teo ZL, Tham YC, Yu M, Chee ML, Rim TH, Cheung N (2021). Global prevalence of Diabetic Retinopathy and Projection of Burden through 2045: systematic review and Meta-analysis. Ophthalmology.

[4] World Health Organization. Diabetic retinopathy screening: a short guide: increase effectiveness, maximize benefits and minimize harm. 2020.

[5] Farley TF, Mandava N, Prall FR, Carsky C (2008). Accuracy of primary care clinicians in screening for diabetic retinopathy using single-image retinal photography. Ann Fam Med.

[6] Williams GA, Scott IU, Haller JA, Maguire AM, Marcus D, McDonald HR (2004). Single-field fundus photography for diabetic retinopathy screening: a report by the American Academy of Ophthalmology. Ophthalmology.

[7] ElSayed NA, Aleppo G, Aroda VR, Bannuru RR, Brown FM, Bruemmer D (2023). Retinopathy, Neuropathy, and Foot Care: standards of Care in Diabetes-2023. Diabetes Care.

[8] Wilkinson CP, Ferris FL, Klein RE, Lee PP, Agardh CD, Davis M (2003). Global Diabetic Retinopathy Project Group. Proposed international clinical diabetic retinopathy and diabetic macular edema disease severity scales. Ophthalmology.

[9] Williams AM, Weed JM, Commiskey PW, Kalra G, Waxman EL (2022). Prevalence of diabetic retinopathy and self-reported barriers to eye care among patients with diabetes in the emergency department: the diabetic retinopathy screening in the emergency department (DRS-ED) study. BMC Ophthalmol.

[10] Raman R, Vasconcelos J, Rajalakshmi R, Prevost A, Ramasamy K, Mohan V (2022). Prevalence of diabetic retinopathy in India stratified by known and undiagnosed diabetes, urban–rural locations, and socioeconomic indices: results from the SMART India population-based cross-sectional screening study. Lancet Glob Health.

[11] Fernandes AG, Ferraz AN, Brant R, Malerbi FK (2022). Diabetic retinopathy screening and treatment through the Brazilian National Health Insurance. Sci Rep.

[12] Avidor D, Loewenstein A, Waisbourd M, Nutman A (2020). Cost-effectiveness of diabetic retinopathy screening programs using telemedicine: a systematic review. Cost Eff Resour Alloc.

[13] Bragge P, Gruen RL, Chau M, Forbes A, Taylor HR (2011). Screening for presence or absence of diabetic retinopathy: a meta-analysis. Arch Ophthalmol.

[14] Price LD, Au S, Chong NV (2015). Optomap ultrawide field imaging identifies additional retinal abnormalities in patients with diabetic retinopathy. Clin Ophthalmol.

[15] Padhy SK, Takkar B, Chawla R, Kumar A (2019). Artificial intelligence in diabetic retinopathy: a natural step to the future. Indian J Ophthalmol.

[16] Hudson SM, Modjtahedi BS, Altman D, Jimenez JJ, Luong TQ, Fong DS (2022). Factors affecting compliance with Diabetic Retinopathy Screening: a qualitative study comparing English and Spanish speakers. Clin Ophthalmol.

[17] Kashim RM, Newton P, Ojo O (2018). Diabetic Retinopathy Screening: a systematic review on patients’ non-attendance. Int J Environ Res Public Health.

[18] Kuo J, Liu JC, Gibson E, Rao PK, Margolis TP, Wilson B (2020). Factors Associated with adherence to Screening guidelines for Diabetic Retinopathy among Low-Income Metropolitan patients. Mo Med.

[19] Lu Y, Serpas L, Genter P, Anderson B, Campa D, Ipp E (2016). Divergent perceptions of barriers to Diabetic Retinopathy Screening among patients and Care providers, Los Angeles, California, 2014–2015. Prev Chronic Dis.

[20] Wong TY, Sabanayagam C (2020). Strategies to Tackle the Global Burden of Diabetic Retinopathy: from epidemiology to Artificial Intelligence. Ophthalmologica.

[21] Shrestha R, Singh P, Dhakwa P, Tetali S, Batchu T, Thapa PS (2023). Augmenting the referral pathway for retinal services among diabetic patients at Reiyukai Eiko Masunaga Eye Hospital, Nepal: a non-randomized, pre-post intervention study. BMC Health Serv Res.

[22] Basch CE, Walker EA, Howard CJ, Shamoon H, Zybert P (1999). The effect of health education on the rate of ophthalmic examinations among African americans with diabetes mellitus. Am J Public Health.

[23] Murray CM, Shah BR (2016). Diabetes self-management education improves medication utilization and retinopathy screening in the elderly. Prim Care Diabetes.

[24] Scanlon PH (2017). The English National Screening Programme for diabetic retinopathy 2003–2016. Acta Diabetol.

[25] Weerasinghe LS, Dunn HP, Fung AT, Maberly G, Cheung NW, Weerasinghe DP (2023). Diabetic Retinopathy Screening at the point of Care (DR SPOC): detecting undiagnosed and vision-threatening retinopathy by integrating portable technologies within existing services. BMJ Open Diabetes Res Care.

